# Inhibition of Return Is Modulated by Negative Stimuli: Evidence from Subliminal Perception

**DOI:** 10.3389/fpsyg.2017.01012

**Published:** 2017-06-20

**Authors:** Fada Pan, Xiaogang Wu, Li Zhang, Yuhong Ou

**Affiliations:** Department of Applied Psychology, School of Education Science, Nantong UniversityNantong, China

**Keywords:** inhibition of return, negative stimuli, subliminal perception, event-related potentials, emotional processing

## Abstract

Inhibition of return (IOR) is considered as a “blindness mechanism” that emotional stimuli have no impact on it. Most previous studies suggested that IOR was not modulated by emotional cues. However, one key question they ignored was that only supraliminal presentation of emotional stimuli was used in their experiments. The present experiment is aimed at exploring the possible interaction between the IOR effect and subliminal emotional process. We manipulated three different kinds of valence strength of negative stimuli (high negative, HN; moderate negative, MN; low negative, LN) which were presented under the subliminal perception level and an event-related potentials (ERPs) recording was adopted. The results showed that, compared to MN and HN, the IOR effect triggered by peripheral cues was more significant for LN with aspects of behavioral and electrophysiological data (a reduction P1 effect, more negative on cued trials than on uncued trials for both early posterior Nd and Nd components). This indicated that IOR can be modulated by emotionally relevant stimuli. The automatic processing that was triggered by subliminally negative stimuli of peripheral cues had an influence on the shifting of spatial attention that was triggered by IOR. These two mechanisms may occur in the perceptual stage simultaneously.

## Introduction

The limited processing capacity of the visual system makes attention select the most valuable information when our visual environment is rich. Moreover, the rapid and efficient attention to stimuli relating to the development or survival of individuals may have a crucial influence on flexible and adaptive behavior, especially for the threatening information. Inhibition of return (IOR) is widely considered as a “foraging facilitation” which contributes to adaptive behavior (Wang and Klein, 2010). Posner and Cohen (1984) first found this phenomenon using a non-predictive cue-target paradigm, combining peripheral cue with the peripheral target. Reaction times are usually faster at the uncued location (cue and target appeared at different positions) than at the cued location when SOA (stimulus onset asynchrony) is more than about 300 ms. The common interpretation is that, by inhibiting attention return to the inspected locations, IOR encourages attention to orient to novel locations. Consequently, the improved efficiency of visual search reflects flexible and adaptive cognitive process (Klein, 2000; Ivanoff and Taylor, 2006). The current experiment is aimed at investigating how the IOR effect will be affected when attention is attracted by biological stimuli (i.e., negative events) during the spatially visual search.

As compared to neutral stimuli, biologically relevant stimuli like threatening events are more sensitive to human behavior (Ohman and Mineka, 2001). If negative events are ignored or over attended, the potentially negative influence of emotional disorder may occur frequently (Bai et al., 2013). In other words, biologically relevant events triggering attentional capture have priority for entering consciousness. If IOR is an evolutionary mechanism of improving search efficiency, its effect size will be affected by biologically relevant events (Silvert and Funes, 2016). However, it is still uncertain that how biological stimuli can modulate IOR effect. Some studies found emotion stimuli can modulate the IOR effect for clinical populations (Fox et al., 2001; Pérez-Dueñas et al., 2014). Other literature measuring the IOR effect of emotional cues or targets, suggests that IOR is a “blindness mechanism” that is not affected by the occurrence of biologically relevant cues and targets (Taylor and Therrien, 2005; Stoyanova et al., 2007; Lange et al., 2008). According to the existing literature, we summarized two mainly reasonable interpretations.

First, different groups of individuals are an important factor. Fox et al. (2001) observed a modulation of IOR for high and low trait-anxious participants. In their experiments, different cue types (happy, angry, and neutral) were presented at the cued location. The IOR effect was eliminated by the angry cues for both high and low trait-anxious participants. The results showed that the trait-anxious participants had difficulty in disengaging their attention from the negative events. Therefore, their capacity of inhibiting the influence of negative events was insufficient. A similar modulation was found in another experiment. Researchers investigated whether real angry face does capture attention among the trait-anxiety and state-anxiety (Pérez-Dueñas et al., 2014). It was clear that the IOR effect was overridden again when targets were biologically angry face than neutral or happy face. Another commonly occurred mental disorder is depression. There are various factors working together for the generation of depression, but attention bias toward the negative events is considered as a key factor. Researchers using emotional cues to explore the IOR effect of depressive individuals found a deficient inhibition of negative events (Dai and Feng, 2009). In summary, based on these studies of the relation between the IOR effect and emotional process among mental disorder individuals, we can conclude that these individuals have an impaired capacity of IOR. They are oversensitive to negative stimuli relative to typical individuals. Once negative stimuli capture their attention, they cannot inhibit the interference of shifting attention to a novel location. Thus, individuals with mental disorders experience more negative emotion.

Second, will emotional process and IOR occur in the same stage (e.g., perceptual stage)? This question may be ignored in most previous literature with non-clinical participants. In a typical cue-target paradigm, IOR is tested during a spatially visual space and it can affect the early visual process of sensory perception (Klein and Dick, 2002). Although the two forms of IOR, motoric and visual, have been acknowledged widely (Taylor and Klein, 2000; Zhao et al., 2011), evidence from cognitive neuroscience studies showed that inhibited amplitudes for the cued location often associated with the IOR effect in the sensory/perceptual ERP components (P1: perceptual; N1, Nd: late-perceptual) (Zhang and Zhang, 2007; Prime and Jolicoeur, 2009; Satel et al., 2014). Those three components are mostly used to investigate IOR effect regardless of types of tasks, time courses or theories, and there is no signal electrophysiological marker for the IOR effect so far (Martín-Arévalo et al., 2015). When researchers use a non-predictive cueing paradigm to investigate the relation between the IOR effect and emotion processing, peripheral cues will trigger the exogenous attentional capture. A bottom-up selective response to potential relevant stimuli is raised from this exogenous capture of attention (Hopfinger and West, 2006). Thus, the IOR effect may mainly reflect the perceptual process of visual search and represents a bottom-up reflexive behavior (Stoyanova et al., 2007; Sapir et al., 2014). One question is that the present time of biologically relevant stimuli in previous literature is hardly less than 200 ms, indicating supraliminal perception. The supraliminal and subliminal visual processes of emotional stimuli have different neural pathways and response characteristics (Bernat et al., 2001). The supraliminal process of emotional stimuli is subject to top-down attentional control (Yamasaki et al., 2002). Thus, this may reflect that the supraliminal perception of emotional stimuli and reflexive IOR may occur in the different stages, leading to no interaction between them. One rational hypothesis of attentional bias should propose that, if attention is captured more easily by biologically relevant stimuli than less relevant stimuli appearing at the cued location, the magnitude of IOR effect will be reduced or eliminated. However, this hypothesis does not fit well with previously experimental results. Whether emotional, neutral faces or spiders are presented at the peripheral cueing locations, the magnitude of IOR effect have no conspicuous difference under all cue conditions (Stoyanova et al., 2007; Lange et al., 2008; Hu et al., 2014), indicating that IOR is blind to emotion stimuli. Another special central cue-target paradigm presented supraliminal emotion cues at the center of the screen found that the attentional system tended to inhibit irrelevant negative emotion but not inhibit irrelevant positive emotion (Chao, 2010). However, researchers recently replicated Chao’s experiment and their reanalysis indicated slower responses to negative facial expressions than positive ones regardless of the preceding cue (Poliakoff et al., 2015). Thus, they thought that orienting in the emotional domain could not be measured by using a cue-target task. On the other hand, manipulation of emotional targets is also used to investigate attentional capture. These studies found the modulation of IOR effect relating with schematic sadly face only in the left visual field (Baijal and Srinivasan, 2011), anxiety induced by threatening context (Rutherford and Raymond, 2010) and schizophrenic patients (Hu et al., 2014, experiment 2). Based on above studies, it seems that IOR is not modulated by emotional cues but emotional targets. Wang et al. (2013) adopted a cue-target paradigm to investigate the IOR process during an emotion recognition task and the underlying mechanisms (event-related potentials, ERPs). Behavioral and electrophysiological results consistently indicated that the attentional bias of emotional targets and IOR occurred in two different stages. Recently, Silvert and Funes (2016) systematically investigated the influence of time courses and task relevance on IOR and the processing of emotionally facial expressions. One important finding is a later IOR effect for fearful faces than neutral faces in an emotion and gender discrimination task. However, this may not reflect the true relation between these two mechanisms. The interaction between emotional target type and cueing type is significant only in short SOA (500 ms) but not in medium (750 ms) or in long SOA (1000 ms). However, SOA shorting than 700–1000 ms is not suitable for the generation of IOR effect (Lupiáñez et al., 1997). From this perspective, the results with medium or long SOA are more reliable. Thus, we incline to the view that supraliminal process of emotional stimuli and IOR may occur in the different stages when more biological stimuli as real angry faces are adapted.

In summary, one definite conclusion can be drawn on that individual differences (mental disorder individuals vs. common individuals) can modulate the IOR effect size. The growth of evidence suggests that a reduced IOR effect is always observed for anxious, schizophrenic, or depressive individuals for their over-attention of negative stimuli (Fox et al., 2001; Pérez-Dueñas et al., 2009, 2014). Another potential question leaving us to explore in the current experiment is whether the subliminal perception of emotion processing can affect the IOR effect. If the subliminal perception of emotion processing triggers a bottom-up selective response, we expect the participants to show an interaction between the IOR effect and emotion processing. Previous studies measuring the effect of subliminal emotion stimuli found that this manipulation of stimuli involved sub-cortex and unconscious process, which leads to a more biologically original response (Van Honk et al., 2001; Hänsel and von Känel, 2013). In addition, subliminal emotion stimuli can draw attention automatically, and then have an impact on spatially attentional orienting (Carlson and Reinke, 2008). To reiterate our hypothesis, if IOR and subliminal emotional processes occur in the same stages (e.g., unconscious stage), there must be some interactions. For this purpose, the current experiment also made some changes in methodological aspects to test our prediction. First, we chose negative stimuli as peripheral cues due to their better evolutionary value (Anderson et al., 2003). Moreover, we further divided them into three different kinds of valence strength (high negative, HN; moderate negative, MN; low negative, LN) (Yuan and Hong, 2012). Second, in order to ensure perception up to subliminal level, we carried out a preliminary experiment before the formal experiment and more details would be presented in the method section. Finally, we used high time resolution of ERPs recording to investigate the early stages of sensory perception.

## Materials and Methods

“This study was approved by the Human Ethics Committee of Nantong University. A written informed consent was obtained from all the participants before their participation”.

### Participant

According to participants’ scores in Hamilton Anxiety Scale (HAMA; Hamilton, 1959), 22 participants (12 female, 10 male; aged 20 to 23 years; scores below 6, having no anxiety) were selected from 35 volunteers to take part in this experiment and gave a reward after participation. All participants had a normal or corrected-to-normal vision, right-handed, without mental illness, color blindness, and hadn’t participated in a similar experiment before.

### Stimuli and Apparatus

Thirty-six photographs were selected from International Affective Picture System (IAPS) (Lang et al., 2008). They were consisted of 30 photographs (each 10 of HN, MN, and LN)^1^ for formal experiment and 6 photographs for a practice block. To ensure the validity of these three kinds of negative pictures, we tested their emotion valence and arousal. We found a significant main effect of emotion valence [*M* ±*SE*: HN = 1.63 ± 0.06, MN = 3.55 ± 0.04, LN = 4.43 ± 0.11; *F*(2,18) = 310.34, *p* < 0.001, η^2^ = 0.97], pairwise comparisons found a significant difference between HN and MN, HN and LN, MN and LN, *p* < 0.001. There was no significant main effect of emotion arousal [*M* ±*SE*: HN = 6.46 ± 0.11, MN = 6.20 ± 0.14, LN = 6.10 ± 0.11; *F*(2,18) = 2.02, *p* > 0.05, η^2^ = 0.18], pairwise comparisons found no significant difference between HN and MN, HN and LN, MN and LN, *p* > 0.05. Pictures converted to grayscale and reduced their visibility down to 10% compared with 100% luminance of original pictures (Mele et al., 2008). We conducted a preliminary experiment to determine the objective threshold of subliminal perception of emotional stimuli. The procedure of preliminary experiment identified with formal experiment, except targets disappearing. A forced-choice task we used to request participants to discriminate emotional valence strength of cues. Number keys of “1,” “2,” and “3” were labeled “HN,” “MN,” and “LN” separately. One-Sample *T*-test of accuracy showed (test value = 0.33) that, when present time of cue was 15 ms, there was no significant difference between accuracy and probability, *p* > 0.1, indicating up to subliminal perception.

E-prime software was used for the presentation of stimuli, data collection on a Dell PC. Each trial started with two rectangle boxes and a fixation. Each box was 2.34° (horizontal) × 3.36° (vertical) and fixation was 0.5° (horizontal) × 0.5° (vertical). Negative stimuli were presented at cue location and a hollow circle was used as the target. Participants sat in a dimly lit and electromagnetically shielded room, with their heads located 70 cm away from the computer screen.

### Design and Procedure

The present experiment consisted of a 2 (cueing: cued vs. uncued) × 3 (cue type: HN, MN, LN) within-subject design. When cue and target appeared at the same location named cued trials, cue and target appeared at different locations named uncued trials. In the course of the experiment, participants performed a localization task as accurately and quickly as possible. Reaction times, accuracy, and EEG data were recorded simultaneously.

**FIGURE 1:**
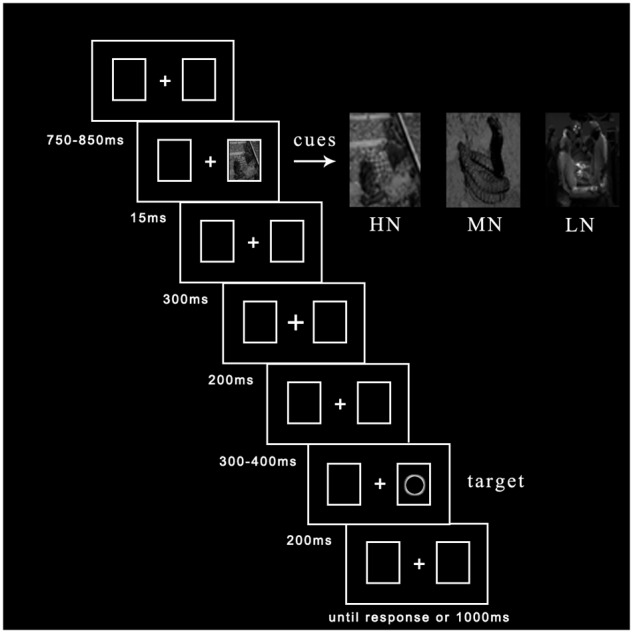
The procedure of events in a sample trial.

To eliminate participants’ tension of mood, we explain fundamental principles of ERP recording before entering the laboratory. Then written informed consent was given them to sign. If they felt bad during the experiment, they could ask to cease experiment at any time. The experimental procedure was depicted in **Figure 1**. Central fixation and two rectangle boxes with a variable duration between 750 and 850 ms were presented at the beginning of each trial. Emotional cues flickered in one of two potential boxes randomly with 50% probability for 15 ms. After 300 ms delay, the fixation cue for 200 ms was presented in the middle box (Prime et al., 2006; Martín-Arévalo et al., 2013; Silvert and Funes, 2016). Subsequently, after a duration delay between 300 and 400 ms, the target (hollow circle) appeared to the left or right box with 50% probability for 200 ms until participants made a response or 1000 ms had elapsed. Participants were asked to judge the location of targets: if target appeared in the left box, please press “Z” key; if target appeared in the right box, please press “M” key. Each trial ended with a black blank for 1500 ms.

A total of 504 randomized trials consisted of 24 practice trials and four blocks of 480 experimental trials. Before experimental blocks, each participant needed to complete practice trials until they understood the experimental requirement and enabled their correct rate up to 95% at least. Each experimental block was composed of 60 trials with cued condition (20 for HN, 20 for MN, 20 for LN) and 60 trials with uncued condition (20 for HN, 20 for MN, 20 for LN). There was the equal probability (50%, non-predictive) for cue and target appeared at left or right, for each one of 30 pictures (16 trials for each picture). The operation of the practice and experimental block was identical. Before the experiment, participants were told to try their best to reduce their actions of the swallow, frown, and blink, to keep their attention on the fixation cross, to keep the body from moving and in the blink of a black blank. Three minutes were provided after every block for a rest.

We interviewed participants whether they could see the pictures after their participation. All participants answered “No,” indicating that they couldn’t be aware of these pictures explicitly.

### ERP Recording and Analysis

Continuous electroencephalogram (EEG) was recorded by a 64 Ag-AgCl electrode elastic cap placed according to the international 10-20 system and the Neuroscan ERP workstation (scan4.5). M1 and GND served as the reference and ground electrode on the left mastoid and medial frontal aspect separately. Vertical electrooculogram (EOG) and Horizontal EOG placed above and below the participant’s left eye and outer canthi of both eyes. The sampling rate and band pass were 1000 Hz/channel and 0.05∼100 Hz. EEG data and behavioral data were recorded simultaneously. Every participant washed their hair in the laboratory first and we started to record until the impedances were stable below 5 KΩ with the conductive paste on the scalp.

After recording, scan software was used for off-line analysis. We merged behavioral data into EEG data and translated the average of M1 and M2 into a new reference. Ocular artifacts like blinks and vertical eye movements were rejected and not included in the construction of ERPs. EEG epoch was -100 ms prior to target onset and 500 ms posterior to target onset containing the rejection of incorrect or no responses trials. After baseline correction, trials with excessive artifacts (±80 μV standard) were rejected for further analysis (acceptance rate of each participant was more than 85% of trials). Finally, mean waveforms under all conditions performed a band-pass filter containing a high-pass filter of 0.1 Hz and a low-pass filter of 30 Hz (24 dB/octave) (Luck, 2014). Data of three participants were excluded from analysis because of excessive EEG artifacts (accepted less than 70% of trials).

According to the overall average map and research literature, the parieto-occipital P1, N1, early posterior Nd (it spans the interval of the P1 and N1 components) and Nd ERP components were analyzed. These ERP components were divided by the time windows in which they occurred: P1, 100∼150 ms; N1, 150∼200 ms; early posterior Nd, 100–200 ms; Nd, 200–300 ms. Based on the previous literature (Prime and Ward, 2006; Prime and Jolicoeur, 2009; Satel et al., 2014), Po7/Po8 were selected for statistical analyses of P1, N1, and Nd. SPSS 16.0 for Windows was used for repeated measures analysis of variance of behavioral data and ERPs data. The *p* of all main effects and interactions were corrected by Greenhouse–Geisser.

## Results

### Behavioral Results

The analysis of RTs was based on the trials with the correct response and was executed within 200–1000 ms first. Then, trials with RTs faster or slower than three standard deviations below or above participants’ mean RTs were excluded. Totally, 4.15% of trials were excluded from statistic analyses of mean RTs and correct rate for each condition.

On correct rate, main effect and interaction were not significant.

**FIGURE 2:**
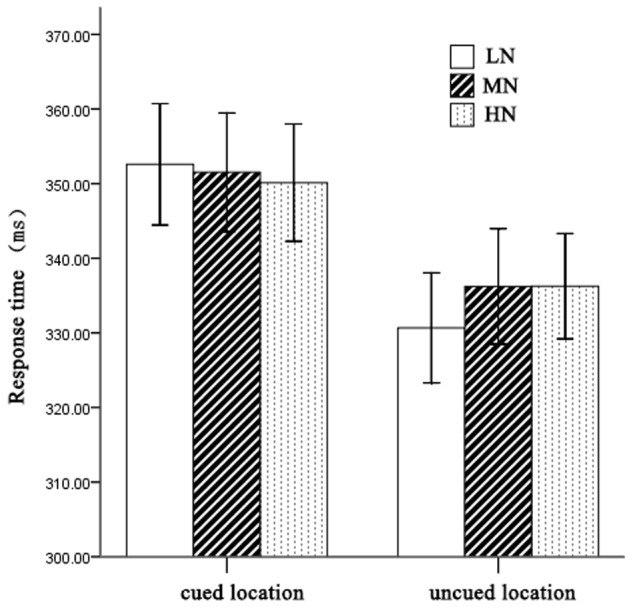
Mean RTs for each condition. Error bars show the SE of each condition.

On mean RTs (see **Figure 2**), 2 (cueing: cued, uncued) × 2 (cue type: HN, MN, LN) repeated measures analysis of variance (ANOVA) revealed a significant main effect of cueing, *F*(1,21) = 33.03, *p* < 0.001, η^2^ = 0.61, indicating faster RTs on uncued trials (334 ± 11 ms) than cued trials (351 ± 12 ms). The main effect of cue type was not significant, *F*(2,42) = 0.62, *p >* 0.05. However, there was a significant interaction between cueing and cue type, *F*(2,42) = 5.94, *p* < 0.01, η^2^ = 0.22. Planned contrast revealed a significant main effect of cue type at uncued location, *F*(2,42) = 3.76, *p* < 0.05, η^2^ = 0.27. *LSD post hoc* test showed that LN (330 ± 11 ms) was faster than MN (336 ± 11 ms) and HN (336 ± 11 ms), *p* = 0.048 and *p* = 0.023 separately. Meanwhile, we compared the IOR effect size (cued-uncued) of HN, MN, and LN. We found the main effect of cue type, *F*(2,42) = 5.94, *p* < 0.01, η^2^ = 0.22, indicating significantly larger magnitudes of IOR for LN (22 ms) than MN (15 ms) and HN (14 ms).

### ERP Results

**FIGURE 3:**
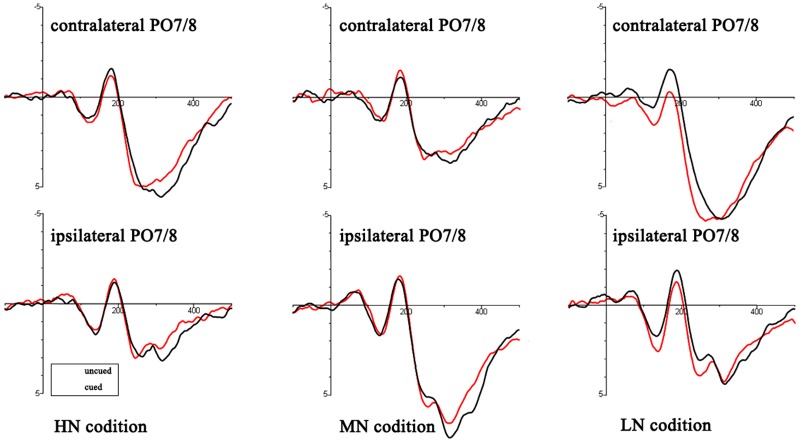
Target-elicited ERP waveforms for HN, MN, and LN trials. The read lines reflect the uncued trials and black lines reflect cued trials.

An ANOVA analysis [peripheral cueing (cued vs. uncued), cue type (HN, MN, LN)] at the time window 0–100 ms showed that (see **Figure 3**): for contralateral electrodes, there was a significant marginally interaction of cueing × cue type, *F*(2,36) = 2.98, *p* = 0.064, η^2^ = 0.14. Planned contrast revealed a significant main effect of cueing at LN condition, *F*(1,18) = 5.99, *p* < 0.05, η^2^ = 0.25, indicating larger amplitudes of uncued trials (0.54 ± 0.29 μV) than cued trials (0.05 ± 0.3 μV); there were no main effects of cueing at MN sand HN. For ipsilateral electrodes, main effects and interactions were non-significant (all *p* > 0.05).

There was a three-factor-repeated-measures ANOVA for mean amplitudes of the P1, N1, and early posterior Nd components: peripheral cueing (cued vs. uncued), cue type (HN, MN, LN), and laterality (ipsilateral vs. contralateral).

P1: The main effect of cue type was laterality, *F*(1,18) = 5.19, *p* < 0.05, η^2^ = 0.22. There was a significant interaction of cueing × cue type, *F*(2,36) = 4.16, *p* < 0.05, η^2^ = 0.19. Planned contrast revealed a significant main effect of cueing at LN condition, *F*(1,18) = 10.62, *p* < 0.01, η^2^ = 0.37, indicating larger amplitudes of uncued trials (2.09 ± 0.51 μV) than cued trials (1.41 ± 0.44 μV); there were no main effects of cueing at MN and HN. Other main effect and interactions were non-significant (all *p* > 0.05).

N1: There was a significant interaction of cueing × cue type, *F*(2,36) = 8.58, *p* < 0.01, η^2^ = 0.32. Planned contrast revealed a significant main effect of cueing at LN condition, *F*(1,18) = 14.23, *p* < 0.01, η^2^ = 0.44, indicating smaller amplitudes of uncued trials (0.22 ± 0.37 μV) than cued trials (-0.72 ± 0.39 μV); there were no main effects of cueing at MN and HN. Other main effect and interactions were non-significant (all *p* > 0.05).

Early posterior Nd: There was a significant interaction of cueing × cue type, *F*(2,36) = 7.56, *p* < 0.01, η^2^ = 0.3. Planned contrast revealed a significant main effect of cueing at LN condition, *F*(1,18) = 17.58, *p* = 0.001, η^2^ = 0.49, indicating larger amplitudes of uncued trials (1.15 ± 0.4 μV) than cued trials (0.4 ± 0.36 μV); there were no main effects of cueing at MN and HN. There was a significant interaction of cueing × laterality, *F*(1,18) = 6.95, *p* < 0.05, η^2^ = 0.28. Other main effect and interactions were non-significant (all *p* > 0.05).

Mean amplitudes of the Nd components was assessed in a three-way, repeated-measures ANOVA with factors for peripheral cueing (cued, uncued), central cueing (indicated, non-indicated), and electrodes (Po7, Po8). The main effect of electrode site was significant, *F*(1,18) = 12.06, *p* < 0.01, η^2^ = 0.4. There was a significant interaction of cueing × cue type, *F*(2,36) = 4.87, *p* < 0.05, η^2^ = 0.21. Planned contrast revealed a significant main effect of cueing at LN condition, *F*(1,18) = 11.34, *p* < 0.01, η^2^ = 0.39, indicating larger amplitudes of uncued trials (4 ± 0.59 μV) than cued trials (3.16 ± 0.62 μV); there were no main effects of cueing at MN and HN. Other main effect and interactions were non-significant (all *p* > 0.05).

Another statistics of planned contrast of EEG data showed that: For P1, N1, Nd, there were no main effects of cue type on cued location but significant main effects of cue type on uncued locations [P1: *F*(2,36) = 8.04, *p* < 0.01, η^2^ = 0.31, the amplitudes of LN (1.93 ± 0.47 μV) were more positive than MN (1.22 ± 0.45 μV) (*p* = 0.001) and HN (1.53 ± 0.45 μV) (*p* = 0.038) separately; N1: *F*(2,36) = 7.51, *p* < 0.01, η^2^ = 0.31, the amplitudes of MN (-0.5 ± 0.36 μV) (*p* = 0.001) and HN (-0.22 ± 0.34 μV) (*p* = 0.052) were more negative than LN (0.22 ± 0.35 μV) separately; Nd: *F*(2,36) = 4.87, *p* < 0.05, η^2^ = 0.21, the amplitudes of LN (4 ± 0.59 μV) were more positive than MN (3.35 ± 0.6 μV) (*p* = 0.009) and HN (3.48 ± 0.59 μV) (*p* = 0.017) separately].

## Discussion

In order to further explore the relation between subliminal emotional process and the IOR effect, we used a location task to examine these two mechanisms from behavioral and electrophysiological aspects. Consistent with our hypothesis, we found that IOR was modulated by three different kinds of valence strength of negative stimuli. There were two main results: First, the IOR effect was modulated by the subliminally emotional process. Second, this modulation occurred in the P1, early posterior Nd and Nd components. This indicates that IOR and subliminally emotional process occurred in perceptual stage simultaneously.

Previous studies used biologically emotional cues usually found no conspicuous difference of the IOR effect (Fox et al., 2001; Stoyanova et al., 2007; Lange et al., 2008; Hu et al., 2014). One key question they ignored was that only supraliminal presentation of emotional stimuli was operated in their experiments. However, emotion processing can occur under unconscious condition (Dolan, 2002; Tamietto and de Gelder, 2010). Smith (2012) found that different kinds of emotional faces including threatening faces could be processed without conscious awareness at early stages. When we asked participants to fix their eyes on central fixation strictly throughout the entire experiment, IOR mainly reflected an input (perceptual) bias (Hilchey et al., 2014). Thus, once a subliminal emotional process occurred, this process occupies the same perceptual pathway as the IOR. We conducted a preliminary experiment to ensure emotional process up to subliminal perception. Compared with MN and HN, the IOR effect of LN was more evident in the behavioral and electrophysiological data. Another study used a cue-target paradigm to explore how the valence intensity of unpleasant stimuli affects subsequent cognitive processing (Yuan et al., 2011). The present time of emotional cues was 1000 ms while participants were asked to discriminate the location of a neutral target. They did not find significant interactions between emotion and validity in the behavioral data and the components of P1, N1. According to our hypothesis, the supraliminal perception of emotional stimuli relating to top-down attentional control (Yamasaki et al., 2002) and reflexive IOR relating to bottom-up response (Stoyanova et al., 2007; Sapir et al., 2014) may occur in different stages, so there is no interaction between them. One reasonable explanation is that subliminal emotional processes may induce attention bias. HN and MN stimuli capture the participants’ attention more easily than LN, thus it is difficult for participants to disengage their attention from the cued location under HN and MN conditions.

An analysis was operated to clarify potential difference at the time window 0–100 ms. The result showed the difference between cue type and cueing effect only at the contralateral electrodes. Especially, we found a reduction 0–100 ms component at the contralateral electrodes. This might indicate the early perceptual inhibition. The current experiment focused on ERP components of perceptual processing (P1 and N1), early posterior Nd and post-perceptual processing (Nd), wherein the oculomotor was suppressed. We all found some difference in these components. The P1 reduced amplitude of cued location than uncued location was usually associated with the IOR effect (McDonald et al., 1999; Prime and Jolicoeur, 2009). For N1, the IOR reflected enhanced amplitude for uncued than cued trials (Prime and Ward, 2004; Prime and Jolicoeur, 2009; Satel et al., 2014). This two diametrically opposed effects on P1 (the P1 ‘reduction’ might not be related to the P1 peak) and N1 (started in the P1 interval) amplitudes reminded us that there may be a single difference between cued and uncued waveforms that spans the time intervals of the P1 and N1 peaks (McDonald et al., 1999; Pan et al., 2017). Thus, the effect of N1 component which appeared to start in the P1 interval needed to be further analyzed (called early posterior Nd in 100–200 ms). The result of early posterior Nd showed that the ERP was more negative on cued trials than on uncued trials of LN, indicating an IOR effect. This early posterior Nd is a signal ERP component that is unrelated to the P1 and N1. The view of perceptual inhibition also found smaller amplitudes of cued location for the early ERP components (Prime and Jolicoeur, 2009), which was fitted well with the result of early posterior Nd. Another alternative explanation was that the early posterior Nd might reflect sensory refractoriness, which could be very common in spatial cueing studies (McDonald et al., 1999). Recently, Nd is considered as a potential neutral marker for IOR, which refers to a reduction of cued location as compared to uncued location (Satel et al., 2013, 2014). However, there was no agreed explanation of the underlying mechanism of Nd. The present experiment also found a reduced Nd component for LN. This indicated that IOR was observed.

In summary, the current experiment found a significant interaction between IOR and subliminal emotional processing, suggesting that those two mechanisms may occur in the same stages simultaneously. As compared to MN and HN, IOR was more significant for LN in behavioral and electrophysiological aspects.

## Author Contributions

FP and XW designed and coordinated the study; XW, LZ, and YO carried out experiments and data process; XW drafted the manuscript; FP reviewed the manuscript. All authors gave the final approval for publication.

## Conflict of Interest Statement

The authors declare that the research was conducted in the absence of any commercial or financial relationships that could be construed as a potential conflict of interest.

## Footnotes

1 Photograph codes: HN: 3053, 3059, 3068, 3100, 3168, 3225, 3266, 9405, 3001, 3102; MN: 1050, 1052, 1090, 1201, 1205, 1300, 1301, 1304, 3022, 6555; LN: 1101, 3211, 5940, 3302, 8475, 4770, 1022, 1321, 1114, 7640.
